# Sarcopenia Prevalence and Risk Factors among Japanese Community Dwelling Older Adults Living in a Snow-Covered City According to EWGSOP2

**DOI:** 10.3390/jcm8030291

**Published:** 2019-02-28

**Authors:** Ya Su, Kengo Hirayama, Tian-fang Han, Miku Izutsu, Michiko Yuki

**Affiliations:** 1 Graduate School of Health Sciences, Hokkaido University, Sapporo 060-0812, Japan; swanvivi@eis.hokudai.ac.jp (Y.S.); idzutsu-m@eis.hokudai.ac.jp (M.I.); 2 Faculty of Health Sciences, Hokkaido University, Sapporo 060-0812, Japan; khirayama@hs.hokudai.ac.jp; 3 Graduate School of Education, Hokkaido University, Sapporo 060-0812, Japan; htf@eis.hokudai.ac.jp

**Keywords:** sarcopenia, prevalence, snow, community dwelling, diabetes, EWGSOP2

## Abstract

Sarcopenia is a common problem among the elderly worldwide. Muscle mass can decrease with aging and decreased physical activity may occur. However, the sarcopenia prevalence among community dwelling older adults living in snow-covered cities remains largely unknown. Therefore, we evaluated the prevalence of and risk factors for sarcopenia in this population aged 65 years or older according to the definitions and diagnoses of the European Working Group on Sarcopenia in Older People-2 from two welfare centers in Sapporo, Hokkaido, Japan. The demographic characteristics, nutrition, and depression status of 310 participants were assessed using a standardized questionnaire. All participants were assessed for grip strength. Skeletal muscle index, body mass index (BMI), and total body water (TBW) were measured using bioelectrical impedance analysis. The overall sarcopenia prevalence in the study population was 8.1%. Multivariate analysis revealed that diabetes and taking more than four drugs per day were independently associated with sarcopenia (adjusted odds ratio (OR) = 3.66, 95% confidence interval (CI) = 1.06–12.61; OR = 2.66, CI = 1.05–6.77, respectively). BMI and TBW were negatively associated with sarcopenia. Sarcopenia prevalence was low in community dwelling older adults living in the snow-covered city. It is indicated that welfare center exercise may be a good intervention for the prevention of sarcopenia. Moreover, the management of diabetes, medication, and nutrition is necessary for sarcopenia prevention in community dwelling older adults.

## 1. Introduction

Sarcopenia is a progressive and generalized skeletal muscle disorder associated with an increased likelihood of adverse outcomes such as falls, fractures, physical disability, and mortality [1]. Patients older than 80 years diagnosed with sarcopenia were found to be three times more likely to report a fall within two years compared with patients without sarcopenia [2]. Patients with sarcopenia tend to have a poor quality of life and greater risk of depression [3,4,5]. In addition, sarcopenia is associated with more frequent hospitalization and cardiovascular events in patients with type 2 diabetes [6]. Previous studies reported two- to three-fold higher sarcopenia prevalence in diabetes patients than in controls [7,8]. Moreover, among community-dwelling older adults, sarcopenia is associated with premature mortality. Community-dwelling older adults in the United States diagnosed with sarcopenia were found to have a 1.29-fold higher risk of all-cause mortality [9].

In Japan, the prevalence of sarcopenia among community-dwelling older adults aged 65–89 years is 21.8% in men and 22.1% in women [10]; in China, the prevalence in older people aged 60 years and older is 10.6% (11.3% in men and 9.8% in women) [11]; and in Korea, the proportions of affected men and women aged 65 years or older are 12.1% and 11.9%, respectively [12]. In Europe, with the definition providing the highest prevalence estimates for sarcopenia, the overall prevalence rates are expected to rise from 20.2% in 2016 to 22.3% in 2045 [13]. On average, 5–13% of people aged older than 60 years have low muscle mass, with the prevalence in those aged older than 80 years increasing to 50% [14].

Aging, cigarette smoking, poor nutritional status, and low BMI (body mass index) as risk factors for sarcopenia are well known [15,16]. In addition to these, many other risk factors are associated with sarcopenia. Polypharmacy is tightly associated with chronic illness and multimorbidity in older adults, and a previous study reported that polypharmacy is associated with clinically relevant sarcopenia [17]. Moreover, some studies reported that a winter environment affects outdoor and physical activities among older adults [18]. The island of Hokkaido, located in the northern part of Japan, has relatively cool summers and snowy winters and is Japan’s coldest region. Sapporo is the largest city in Hokkaido and is snow-covered for a long period during winter during which older people may have difficulty participating in outdoor activities and may have reduced physical activities because of the snow covering. Moreover, fear of falling could be an important factor contributing to decreased outdoor physical activity for older adults [19]. Several research studies have reported the prevalence of sarcopenia in community dwelling older adults using various diagnostic criteria. However, as no research studies have evaluated the prevalence of sarcopenia in community dwelling adults living in a snow-covered city, this particular prevalence remains largely unknown. The purposes of this study were to evaluate the prevalence of and associated factors for sarcopenia among community dwelling older adults living in Sapporo using the updated definition and diagnostic criteria of the European Working Group on Sarcopenia in Older People (EWGSOP2).

According to the EWGSOP2, sarcopenia has low prevalence (8.1%) in Japanese community dwelling older adults living in the snow-covered city of Japan. The prevalence rates of sarcopenia among men and women are 10.1% and 7.2%, respectively. Diabetes and taking more than four drugs per day were independently associated with sarcopenia. Furthermore, BMI and TBW (total body water) were negatively associated with sarcopenia among Japanese community dwelling older adults.

## 2. Materials and Methods

### 2.1. Study Design and Population

This study is part of a large survey: The Nutritional Status of Japanese Community dwelling Older People. This nutritional survey is a cross-sectional study that aims to investigate the living conditions, diet, and nutrition of Japanese community dwelling older people in order to improve their health, functional performance, and quality of life. Sapporo is cold and snowy in the winter, with the roads covered with snow from November to April, therefore older adults might have reduced levels of physical activity. Thus, the survey population was recruited in August 2018 in Sapporo, Hokkaido, Japan, from regular attendees of two welfare centers (a social facility for elderly people). Participants were aged older than 65 years.

Participants who met the following criteria were included; (1) aged 65 years or older; (2) able to walk without help; (3) willing to complete the survey; and (4) provided consent to participate. Trained interviewers administered a standardized questionnaire to collect information for the study.

### 2.2. Ethical Approval for Studies and Informed Consent

All the procedures performed in this study were in accordance with the Declaration of Helsinki and were approved by the Ethics Committee of the Faculty of Health Sciences, Hokkaido University (Reference No 18-22-1). Each participant signed an informed consent document after receiving a detailed verbal explanation of the study objectives.

### 2.3. Demographic Characteristics

Demographic characteristics included age, sex, family composition, smoking status, alcohol consumption, prescription drug use, and medical conditions. Medical conditions included hypertension and diabetes, for which they self-reported their daily medication.

### 2.4. Nutrition and Depression Assessment

To assess the nutritional status of the participants, the Mini Nutritional Assessment Short Form (MNA^®^-SF) was used. The MNA^®^-SF, which comprises six questions, included decline of food intake, weight loss in the last three months, mobility, actual disease/distress in the last three months, neuropsychological problems, and additional anthropometric measures (body mass index or calf circumference). For our study, body mass index (BMI) was used as measured by bioelectrical impedance analysis (BIA). The scores of the short-form MNA^®^ are summed to give a total of 0–14; scores of ≥12 indicate a normal nutritional status, scores from 8 to 11 indicate a risk of malnutrition, and scores of ≤7 indicate malnutrition [20]. The MNA^®^-SF has been shown to be a valid nutrition screening tool for use in community dwelling older adults [21].

The Geriatric Depression Scale (GDS-15) was used to assess depression states among the participants. This is a short version which comprises 15 questions, and the total scores are in the range 0–15; scores of >5 were considered to indicate depression [22]. The GDS-15 is a reliable and valid screening tool for major depression in community living Asian older adults [23].

### 2.5. Muscle Strength

Grip strength, as a measure of muscle strength, was measured using a hand-held dynamometer (MCZ-5041; Macros, Tokyo, Japan) as grip strength is a reliable measure of muscle strength and is easy to measure [1]. Both hands were measured with all the participants standing and the interphalangeal joint of the index finger was positioned at a 90° angle. The measurement was repeated two times for each hand, and the average of the maximum value in either hand was used for the analyses. According to the suggestion of the EWGSOP2, low muscle strength (low handgrip strength) was defined as <27 kg for men and <16 kg for women [1].

### 2.6. Anthropometry

Height and weight were measured using a stadiometer and a weight-measuring instrument. Then, body composition was measured using bioelectrical impedance analysis (BIA) (InbodyS10, Biospace, Korea) at 50 kHz with a seated posture for participants who have no pacemaker implantation. The participants rested for approximately 10–15 minutes before the test, arms were posed lowered naturally, and thighs did not touch each other but were spread to shoulder width. The body composition measurement included body fat percentage (BF%), body mass index (BMI), total body water (TBW), and Skeletal Muscle Mass Index (SMI). The SMI was defined as appendicular skeletal muscle divided by height squared in meters (ASM/height^2^) [24]. According to the suggestion of the EWGSOP2, SMI values of <7.0 kg/m^2^ for men and <6.0 kg/m^2^ for women were defined as low muscle quantity or quality [1].

### 2.7. Diagnostic Criteria for Sarcopenia

Sarcopenia was defined as low muscle strength (low handgrip strength) plus low muscle quantity or quality (SMI: ASM/height^2^) according to the EWGSOP2 [1]. Sarcopenic obesity is defined as sarcopenia in combination with obesity. Obesity is defined as BF% ≥35% in women and BF% ≥28% in men [25].

### 2.8. Sample Size

The sample size was calculated according to formula $n=\frac{Z^{2}\;P\;\left(1-P\right)}{d^{2}}$, Z is the statistic for a level of confidence, as the results presented with 95% confidence intervals (CI), which is conventional, the *Z* value is 1.96 [26,27]. *p* is the estimated prevalence of sarcopenia and is equal to 0.20 based on the prevalence of previous studies among community dwelling older adults [10], and *d* is precision, here chosen to be 0.05 [26]. As a result, the target sample size equaled 246, based on $n=\frac{1.96^{2}\;0.2\;\left(1-0.2\right)}{0.05^{2}}$, considering the 20% of no response rate, thus, the final desired sample size was 295. According to the final desired sample size, the study actually investigated a total of 310 participants.

### 2.9. Statistical Analyses

All data analyses were conducted using IBM SPSS Statistics Version 22.0 (IBM, Armonk, NY, USA). Regarding descriptive statistics, continuous variables were presented using mean and standard deviations and categorical variables using frequencies and percentages. Additionally, the chi-squared test and Fisher’s exact test were used for the categorical variables, whereas a *t*-test was used for the continuous variables, with a normal distribution used to analyze subgroups according to sex. We also used a two-way analysis of variance to analyze the association between age group and sex on Mini Nutritional Assessment Short Form (MNA-SF) scores in the participants. The relationship between sarcopenia and its potentially associated factors was then estimated by deriving odds ratios (ORs) and 95% confidence intervals (CIs) from a univariate logistic regression model. Finally, variables with *p* ≤ 0.20 in the univariate logistic regression model were entered into the multivariate logistic regression model. The model was constructed with stepwise and forward elimination algorithms used to identify the independently associated factors of sarcopenia. *p*-values of <0.05 were considered statistically significant. Goodness-of-fit for multivariate logistic regression models was assessed using the Hosmer-Lemeshow test (H-L test). We also checked the multicollinearity among variables using the variance inflation factor shown in Table A1.

## 3. Results

### 3.1. Participant Characteristics

We included 310 participants with a mean age of 76.0 ± 5.8 years (65–91 years); 221 (71.3%) were women. A total of 303 participants had body composition measured by BIA; seven participants without sarcopenia could be not measured because of pacemaker implantation. The characteristics of the study population according to sex are presented in Table 1. The men were significantly older than the women (mean age: 77.4 ± 5.7 vs. 75.4 ± 5.8 years), and more women than men lived alone. Not surprisingly, smoking, consuming alcohol, BMI, TBW, SMI, and grip strength were significantly lower in the women than in the men (Table 1).

### 3.2. Nutrition and Depression Status

**Figure 1:**
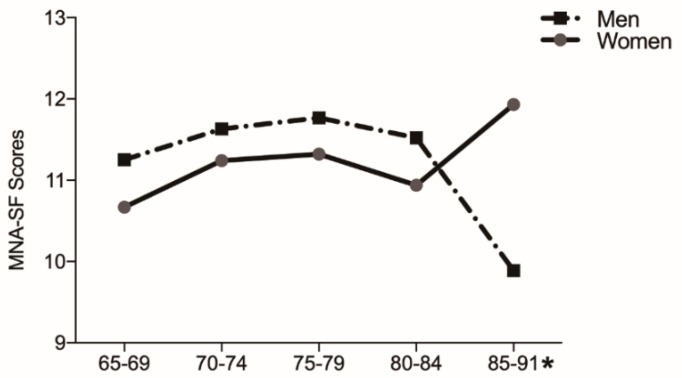
The Mini Nutritional Assessment Short Form (MNA-SF) scores for each sex and age group. *: MNA-SF scores showed significant differences for each sex in the 85–91-year age group.

Only half of the older adults were found to have a normal nutritional status in the entire study population, with the prevalence rates of malnutrition risk and malnutrition being 47.7% and 2.3%, respectively (Table 1); however, nutritional status was not statistically significantly different between women and men (*p* = 0.163). According to age group, the men’s MNA-SF scores were significantly lower than those of the women in the 85–91-year age group (*p* = 0.003; Figure 1). Forty-eight participants were considered depression by GDS-15, the prevalence of which was 15.7% in men and 15.4% in women (*p* = 0.939; Table 1).

### 3.3. Sarcopenia Prevalence

**Figure 2:**
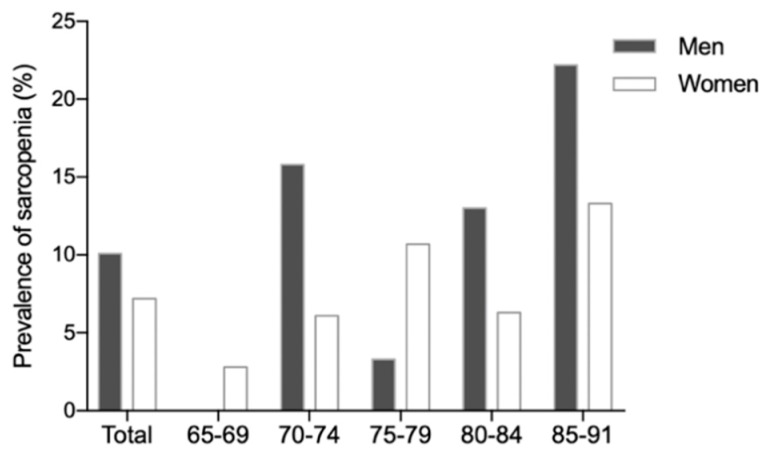
The prevalence of sarcopenia for each sex and each age group.

A total of 45 (14.5%) participants had probable sarcopenia (low handgrip strength), and all participants with probable sarcopenia were measured for muscle quantity (SMI) by BIA. The prevalence of sarcopenia was 8.1% among the community dwelling participants, with no significant difference between men and women (10.1% vs. 7.2%, *p* = 0.401), (Table 1). Figure 2 shows the prevalence of sarcopenia for each sex and age group (*p* = 0.335). Seventy-nine (25.5%) participants had obesity, and only two participants had sarcopenic obesity (Table 2). The characteristics of participants with sarcopenia according to gender are shown in Table A2.

### 3.4. Associated Factors for Sarcopenia

The potentially associated factors were selected from the univariate logistic regression (Table 2), and those factors with *p* ≤ 0.20 were entered into the multivariate logistic regression model (Table 3). The H-L test of multivariate logistic regression gave *p* = 0.600. After adjusting for age, nutritional state, smoking, consuming alcohol, obesity, diabetes, and taking more than four prescription drugs per day were found to be independently associated with sarcopenia (OR, 3.66, 95% CI, 1.06–12.61; OR, 2.66, 95% CI, 1.05–6.77, respectively). Furthermore, BMI and TBW were found to be negatively associated with sarcopenia as defined by the EWGSOP2 (OR, 0.75, 95% CI, 0.62–0.90; OR, 0.84, 95% CI, 0.73–0.96, respectively).

## 4. Discussion

Sarcopenia is a major clinical and public health problem in older people for which geriatricians and scientists in Europe and Asia have developed a consensus definition and diagnostic criteria for sarcopenia that is used worldwide. In 2010, the EWGSOP first proposed consensus guidelines for a clinical definition and diagnosis criteria for sarcopenia [28]. In 2011, the International Working Group on Sarcopenia published its consensus definition of sarcopenia [29]. In 2014, the Asian Working Group for Sarcopenia (AWGS) published regional guidelines to promote research on sarcopenia in Asia [30]. Finally, in 2018, the EWGSOP updated the definition and diagnosis of sarcopenia, producing the EWGSOP2, which was published in the journal Age and Ageing [1].

Following the EWGSOP2, we found that the sarcopenia prevalence rates in Japanese community dwelling older adults (aged 76.0 ± 5.8 years) among men and women were 10.1% and 7.2%, respectively. Two previous studies described the prevalence of sarcopenia in Japanese community dwelling older adults. One study involving 1882 older adults (aged 74.9 ± 5.5 years) was conducted in Kyoto, and one involving 1158 (aged 74.4 ± 6.4 years) was conducted in Osaka, Japan. Sarcopenia as defined by the EWGSOP criteria in both of these studies had prevalence rates among older adults of 21.8% and 22.1% in men and women, respectively, and 11.3% and 10.7% in men and women, respectively [10,31]. We also found several studies from other countries in Asia. The prevalence of sarcopenia among Thai community dwelling elders was 30.5% [32]; its prevalence rates in the Chinese according to the AWGS definition among men and women were 11.3% and 9.8%, respectively, and its prevalence rates in the Korean population among men and women were 12.1% and 11.9%, respectively, according to ASM/weight [9,10]. Although the participants of these studies were community dwelling older adults, the prevalence rates among them are difficult to compare because of the different inclusion criteria for populations and different definitions, measurements, and cutoff values considered. In addition to these, the winter climate in Sapporo is cold and snowy. In particular, the city if snow-covered for a long period of time during winter; therefore, older adults may have reduced physical activity because of the snow covering. Prior studies have reported that cold conditions are associated with decreased physical activity [33]. However, it is worth mentioning that this study indicated a low prevalence (8.1%) of sarcopenia among the community dwelling older population living in the snow-covered city. This is perhaps for the following reasons. The population in this study was taken from regular attendees of welfare centers, so most participants were independent and healthy; in addition, they usually participate in physical activities or exercise at the welfare center. A previous study found that the prevalence of sarcopenia using the IWGS criteria was lower than that using the EWGSOP [34]. There are few studies reporting the prevalence of sarcopenia using EWGSOP2 because of the new definitions but one study showed that prevalence according to EWGSOP2 appears to decrease compared to that according to EWGSOP (2010) [35]. Furthermore, in this study, we found only two participants with sarcopenic obesity. A cohort study conducted in South Korea indicated that the prevalence of sarcopenic obesity ranged from 1.3% to 15.4% in men and from 0.8% to 22.3% in women among healthy volunteers (aged 20–80 years) [36]. It is indicated that welfare center exercise may be a good intervention for the prevention of sarcopenia and sarcopenic obesity. Future research should investigate whether reduced physical activity in cold regions is responsible for decreased muscle mass among community dwelling older adults.

Only half of the older adults in our study had a normal nutritional status, although no significant difference was found between nutritional status and sarcopenia. Additionally, the men’s MNA-SF scores in the age range of 85–91 years were significantly lower than those of women in the same age group; this could be attributable to the difficulty that men aged 85–91 years living alone have in achieving a diverse food intake. Previous studies reported that approximately 50% of urban Japanese older adults ate alone, which has been associated with lower BMI and depression. Eating alone has also been associated with lower food diversity, particularly for men [37,38]. A 4-year prospective study found that dietary variety was associated with grip strength and usual gait speed. Future studies should investigate the relationship between dietary variety and sarcopenia [39].

Several studies have reported that those with sarcopenia are more likely to be depressed [5,40,41]. However, there was no association between sarcopenia and depression among the community dwelling older population in our study, perhaps because the research population frequently participated in exercise and conversation at the welfare center. A previous review found that age, sex, and BMI are the risk factors most associated with sarcopenia among community dwelling older adults [42]. One study found that the prevalence of sarcopenia was increased in women aged 65–74 years and in men aged older than 85 years [8]. Some studies reported a higher prevalence of sarcopenia in men than in women [34,43], whereas another indicated that women have a higher risk [44]. However, in our study, neither sex nor age was found to show a difference in sarcopenia prevalence. We found that lower BMI is a risk factor for sarcopenia, which is consistent with the results of previous studies (OR, 0.75; 95% CI, 1.12–12.65) [43,45]. We also found that low TBW is an associated risk factor for sarcopenia, with an adjusted OR of 0.84 and a 95% CI of 1.12–12.65. Water constitutes an important part of the human body, comprising approximately 75% of skeletal muscle. A previous study reported that body water could be lost with age in both men and women [46]. However, relatively few studies have reported the relationship between TBW and sarcopenia.

One of the most important findings in our study was that community dwelling older adults who take more than four prescription drugs per day had a 2.66-fold higher risk of sarcopenia than participants who took fewer than four prescription drugs per day. Previous studies also reported that taking five or more drugs is a risk factor for sarcopenia, frailty, disability, and mortality in community dwelling older adults and for falls in outpatients [18,47,48]. As a matter of course, the greater the number of medications taken, the greater the risk of adverse drug reactions (ADR) and probability of potential drug–drug interactions [49,50]. In addition, some drugs could interfere with various metabolic processes in the body. Increasing the number of medications taken by older adults was also associated with poorer nutritional status because of medication-associated gastrointestinal or other side effects; for example, the intake of fiber, fat-soluble vitamins, B vitamins, carotenoids, and minerals was lower in elderly with an increased number of medications [51]. Moreover, other side effects of the medications used may be associated with adverse redistribution of muscle and fat. For example, drug-induced orthostatic hypotension may discourage people from physical activity; moreover, the likelihood of sedentariness has been shown to increase with every additional medication prescribed [52]. Furthermore, drug-induced mitochondrial dysfunction, lack of blood flow to muscle, and hormonal and electrolyte disorders probably account for polypharmacy-associated sarcopenia [14]. Considering the possible association between polypharmacy and sarcopenia, medication management for older people might be helpful in preventing future sarcopenia.

This study also found diabetes to be significantly associated with an increased risk of sarcopenia, with an adjusted OR of 3.76 and a 95% CI of 1.12–12.65. Diabetes is a chronic disease characterized by disorders in insulin secretion. Given that muscle is the primary tissue involved in glucose disposal, insulin receptors in the muscle are an important factor in glucose regulation. Insulin stimulates protein and muscle synthesis; thus, impairments in insulin signaling could lead to insulin resistance, affecting muscle synthesis. Insulin resistance is common in obesity, type 2 diabetes, and other diseases, and might be one of the mechanisms involved in sarcopenia induction [53]. Previous studies showed that sarcopenia prevalence was higher in patients with type 2 diabetes than in controls [54,55]. Furthermore, sarcopenia with diabetes was associated with high mortality in men and hospitalization in men and women [56]. Future studies are required to determine the mechanisms of sarcopenia in patients with type 1 and type 2 diabetes mellitus.

Multiple factors lead to sarcopenia. These findings suggest that diabetes management and medication management play an important role in the prevention of sarcopenia among the community dwelling older population. Moreover, a low prevalence of normal nutritional status was observed among the entire study population, even though Japan is a high-income country. This emphasizes the importance of routine screening and early detection of nutritional risks and requirements, as well as the development of effective interventions to ensure that older adults have adequate nutritional status.

There are some limitations in this study that should be considered in future studies. First, this was a cross-sectional study using data collected at a single point in time to examine the relationship between sarcopenia and risk factors. Further research should comprise a longitudinal design to better understand the development and outcomes of sarcopenia with these risk factors among a community dwelling older population living in a snow-covered city. Second, we did not measure physical performance; therefore, people with severe sarcopenia were not identified in the present study. Further research is required to identify those with severe sarcopenia and to clarify the risk factors involved. Finally, the entire study population comprised regular attendees of welfare centers; therefore, most participants were independent and may not represent the general older Japanese population.

## 5. Conclusions

Sarcopenia prevalence is low among community dwelling older adults living in the snow-covered city of Japan, with men and women showing no significant difference in prevalence. Only 50% of older adults had a normal nutritional status. In addition, the MNA-SF scores of men aged 85–91 years were significantly lower than those of women of the same age group. Diabetes, taking more than four prescription drugs per day, low BMI, and low TBW were found to be associated risk factors for sarcopenia. It is indicated that welfare center exercise may be a good intervention for the prevention of sarcopenia. Furthermore, intervention studies examining the effect of diabetes, medication, and nutrition management for the prevention of sarcopenia are required in the future.

## Figures and Tables

**Table 1:** Characteristics of the study participants according to sex.

Characteristics	Total *n* = 310	Men *n* = 89	Women *n* = 221	*p*-Value
Age (years)	76.0 ± 5.8	77.4 ± 5.7	75.4 ± 5.8	0.005 *
Living alone, *n* (%)	110 (35.5)	19 (21.3)	91 (41.2)	0.001 *
GDS-15 (>5), *n* (%)	48 (15.5)	14 (15.7)	34 (15.4)	0.939
Nutritional status, *n* (%)				0.163
Malnutrition risk	148 (47.7)	35 (39.3)	113 (51.1)	
Malnourished	7 (2.3)	2 (2.2)	5 (2.3)	
Current comorbidities, *n* (%)				
Diabetes	26 (8.4)	9 (10.1)	17 (7.7)	0.487
Hypertension	79 (25.5)	21 (23.6)	58 (26.2)	0.628
Take >4 prescription drugs/day	96 (31)	32 (38.2)	62 (28.1)	0.080
Smoking, *n* (%)				<0.001 *
Current	22 (7.1)	12 (13.5)	10 (4.5)	
Former	76 (24.5)	48 (53.9)	28 (12.7)	
Consumes alcohol, *n* (%)	119 (38.4)	47 (52.8)	72 (32.6)	0.001 *
BMI (kg/m^2^)	22.7 ± 2.9	23.3 ± 3.1	22.5 ± 2.8	0.018 *
TBW (L)	28.7 ± 5.2	35.1 ± 4.1	26.2 ± 2.8	<0.001 *
SMI (kg/m^2^)	6.4 ± 0.9	7.4 ± 0.8	6.0 ± 0.6	<0.001 *
Obesity, *n* (%)	79 (25.5)	25 (31.6)	54 (68.4)	0.503
Handgrip strength (kg)	24.5 ± 7.2	32.0 ± 6.7	21.5 ± 4.9	<0.001 *
Sarcopenia, *n* (%)	25 (8.1)	9 (10.1)	16 (7.2)	0.401

Results are presented as mean ± SD, or *n* (%). GDS: geriatric depression scale; BMI: body mass index; TBW: total body water; SMI: skeletal muscle index. *: *p* < 0.05.

**Table 2:** Univariate logistic regression of the potentially associated risk factors for sarcopenia.

Variables	Sarcopenia as Defined by EWGSOP2		
	Total *n* = 25	Univariate Model OR (95% CI)	*p*-Value
Age (years)	77.8 ± 5.5	1.06 (0.99–1.14)	0.100
Female	16 (64.0)	0.69 (0.30–1.63)	0.403
Living alone	9 (36.0)	1.03 (0.454–2.40)	0.955
GDS-15 (>5)	2 (8.0)	0.44 (0.10–1.93)	0.275
Nutritional status			0.164
Normal	13 (52)	1.0 (reference)	
Risk of malnutrition	10 (40.0)	0.79 (0.34–1.87)	0.590
Malnutrition	2 (8.0)	4.37 (0.77–24.78)	0.096
Current comorbidities			
Diabetes	6 (24.0)	4.18 (1.50–11.66)	0.006 *
Hypertension	7 (28.0)	1.20 (0.46–2.87)	0.763
Takes >4 prescription drugs/day	13 (52.0)	2.64 (1.15–6.02)	0.021 *
Smoking			0.111
Never	18 (72)	1.0 (reference)	
Current	4 (16.0)	2.40 (0.73–7.84)	0.149
Former	3 (12)	0.45 (0.13–1.55)	0.202
Consumes alcohol	5 (20.0)	0.38 (0.14–1.03)	0.057
BMI	20.5 ± 1.9	0.72 (0.60–0.85)	<0.001 *
TBW	25.2 ± 3.2	0.80 (0.70–0.90)	0.001 *
Obesity	2 (0.08)	0.23 (0.05–0.99)	0.048 *

Results are presented as mean ± SD or *n* (%). OR: odds ratio; CI: confidence interval; GDS: geriatric depression scale; BMI: body mass index; TBW: total body water. *p*-values in bold represent values <0.05; *: *p* < 0.05.

**Table 3:** Multivariate logistic regression for the associated risk factors for sarcopenia.

Variables	Sarcopenia Defined by EWGSOP2	
	Multivariate Model OR (95% CI)	*p* Value
Diabetes	3.66 (1.06–12.61)	0.040
Takes >4 prescription drugs/day	2.66 (1.05–6.77)	0.040
BMI	0.75 (0.62–0.90)	0.003
TBW	0.84 (0.73–0.96)	0.013

Age, nutritional state, smoking, consuming alcohol, and obesity were adjusted for. OR: odds ratio; BMI: body mass index; TBW: total body water. Goodness-of-fit: H-L Chi^2^ (8) = 6.423, *p* = 0.600.

## Appendix A

**Table A1:** The results of multicollinearity.

Variables	Tolerance	VIF
Age (years)	0.953	1.050
Nutritional status	0.948	1.055
Diabetes	0.936	1.069
Takes >4 prescription drugs/day	0.939	1.065
Consumes alcohol	0.934	1.071
BMI	0.477	2.098
TBW	0.654	1.530
Obesity	0.554	1.805

VIF: variance inflation factor; BMI: body mass index; TBW: total body water.

**Table A2:** Characteristics of the sarcopenia participants according to sex.

Characteristics	Total *n* = 25	Men *n* = 9	Women *n* = 16	*p*-Value
Age (years)	77.8 ± 5.5	78.6 ± 6.2	77.4 ± 5.3	0.620
Living alone, *n* (%)	9 (36.0)	4 (44.4)	5 (31.3)	0.671
GDS-15 (>5), *n* (%)	2 (8.0)	1 (11.1)	1 (6.3)	1.000
Nutritional status, *n* (%)				0.234
Malnutrition risk	10 (40.0)	3 (33.3)	7 (43.8)	
Malnourished	2 (8.0)	2 (22.2)	0 (0.0)	
Current comorbidities, *n* (%)				
Diabetes	6 (24.0)	1 (33.3)	3 (18.8)	0.630
Hypertension	7 (28.0)	1 (11.1)	6 (37.5)	0.355
Take >4 prescription drugs/day	13 (52.0)	5 (55.6)	8 (50.0)	1.000
Smoking, *n* (%)				0.003 *
Current	4 (16.0)	3 (33.3)	1 (6.3)	
Former	3 (12.0)	3 (33.3)	0 (0.0)	
Consumes alcohol, *n* (%)	5 (20)	2 (22.2)	3 (18.8)	1.000
BMI (kg/m^2^)	20.5 ± 1.9	20.9 ± 1.8	20.3 ± 2.0	0.426
TBW (L)	25.1 ± 3.2	28.6 ± 2.5	23.3 ± 1.6	<0.001 *
SMI (kg/m^2^)	5.7 ± 0.6	6.2 ± 0.5	5.4 ± 0.3	<0.001 *
Obesity, *n* (%)	2 (8.0)	1 (11.1)	1 (6.3)	1.000
Handgrip strength (kg)	16.6 ± 6.1	23.9 ± 1.8	12.5 ± 3.0	<0.001 *

Results are presented as mean ± SD or *n* (%). GDS: geriatric depression scale; BMI: body mass index; TBW: total body water; SMI: skeletal muscle index. *: *p* < 0.05.
